# The fibularis tertius muscle revisited: comparative anatomy, developmental classification, and evolutionary-clinical implications

**DOI:** 10.3389/fcell.2025.1647572

**Published:** 2025-08-29

**Authors:** Łukasz Olewnik, Ingrid C. Landfald, Daria Domosławka, George Triantafyllou, Georgi P. Georgiev, Maria Piagkou

**Affiliations:** ^1^ Department of Clinical Anatomy, Mazovian Academy in Płock, Płock, Poland; ^2^ VARIANTIS Research Laboratory, Mazovian Academy in Płock, Płock, Poland; ^3^ Department of Anatomy, Faculty of Medicine, National and Kapodistrian University of Athens, Athens, Greece; ^4^ Department of Orthopedics and Traumatology, University Hospital Queen Giovanna ISUL, Medical University Sofia, Sofia, Bulgaria

**Keywords:** fibularis tertius, peroneus tertius, anatomical variation, fetal development, tendon insertion, lateral ankle instability, bipedalism, superficial fibular nerve

## Abstract

The fibularis tertius muscle (FTM) has long been regarded as an accessory or vestigial structure with inconsistent presence in the human population. However, recent anatomical, developmental, and biomechanical evidence suggests that FT is a functional and evolutionarily novel muscle with distinct clinical implications. This review synthesizes current knowledge on FT morphology, embryological development, phylogenetic context, imaging challenges, and surgical relevance. A unified classification of its proximal and distal attachments is proposed for both fetal and adult populations. The clinical significance of FT variants, including agenesis and fusion with adjacent structures, is examined in the context of tendon harvesting, lateral ankle stabilization, and nerve assessment. Additionally, future directions are outlined, including fetal imaging, biomechanical modeling, and comparative EMG studies, aiming to integrate FT more comprehensively into anatomical atlases, radiological protocols, and surgical planning.

## 1 Introduction

### 1.1 Overview of the fibularis tertius muscle as a traditionally overlooked and variably present muscle of the anterior leg compartment

The fibularis tertius muscle (FTM) is a small, frequently variable muscle situated in the anterior compartment of the leg, classically described alongside the tibialis anterior, extensor hallucis longus, and extensor digitorum longus (Moore et al., 2017; Standring, 2016). Among these, the FTM is the most superficially located and often the least emphasized in anatomical education. Its prevalence varies widely across populations and study methodologies, with values ranging from 38% to nearly 100% (Palomo-López et al., 2019; Yammine and Eric, 2017). It typically originates from the distal portion of the fibula and anterior intermuscular septum and inserts into the fifth metatarsal, contributing to dorsiflexion and eversion (Olewnik, 2019; Rourke et al., 2007).

### 1.2 Historically described as “accessory” or “vestigial”; often omitted from atlases

Historically, the FTM has been labeled as an accessory or vestigial muscle with minimal functional significance (Jungers et al., 1993). It is inconsistently illustrated in anatomical atlases and often absent from clinical considerations (Lambert, 2016; Pośnik et al., 2023). This perception stems partly from its variable presence and reduced prevalence in non-human primates (Kimura and Takahashi, 1985), leading to the assumption of evolutionary regression. However, more recent studies highlight its relevance in stabilizing the lateral column of the foot, preventing hyperinversion, and contributing to functional gait mechanics (Iceman et al., 2020; Jungers et al., 1993; Salem et al., 2018).

### 1.3 Objective: to provide a comprehensive synthesis of the FTM’s embryology, adult morphology, comparative anatomy, imaging features, and clinical relevance

Despite longstanding neglect, a growing body of evidence suggests the FTM plays a more integral role in both human evolution and clinical anatomy than previously appreciated. Embryologically, the FTM likely arises from the deep extensor mass that migrates proximally during lower limb development. Classic studies by Bardeen and Lewis (Bardeen and Lewis, 1901) noted the early distinction of extensor subdivisions in embryos measuring 14–20 mm, with the FTM presumed to develop either independently or as a derivative of the extensor digitorum brevis. More recent developmental imaging confirms this variability and supports its functional specialization in humans (Diogo et al., 2019).

The aim of the present review is to synthesize and critically analyze current knowledge of the FTM. This includes its embryological origins, morphological variability in both adult and fetal populations, comparative anatomy across primates and other mammals, imaging characteristics via ultrasound and MRI, and its diverse clinical implications ranging from surgical relevance to pathologies such as tendon tears, impingement, and transfer procedures. Special attention is given to classifications proposed by Olewnik (2019), which offer a structured framework for understanding this complex and often underestimated muscle.

## 2 Developmental anatomy

### 2.1 Proximal tendon development in human fetuses

The proximal development of the FTM in human fetuses displays significant morphological diversity, indicating early ontogenetic plasticity. According to Ruzik et al. (2022), 100 lower limbs (from 50 spontaneously aborted human fetuses aged 18–38 gestational weeks) were dissected bilaterally and examined. The authors proposed a four-type classification system based on the specific site of the proximal attachment along the fibula and its fascial relations:

• Type I: Originating from the proximal third of the fibula and the anterior intermuscular septum. This type was relatively rare and observed in 5 specimens.
• Type II: Arising from the middle third of the fibula and intermuscular septum. This was the most common configuration, observed in 21 cases, suggesting that this origin may represent the default developmental trajectory.
• Type III: Characterised by the absence of a distinct muscle belly, with the tendon emerging directly as a fascial slip from the extensor digitorum longus. This type was found in 8 limbs and is thought to reflect secondary differentiation from the deep extensor stratum, possibly via a remnant of the extensor digitorum brevis complex (Diogo et al., 2019).
• Type IV: Originating from the distal third of the fibula and the intermuscular septum. This variant was identified in 16 cases and closely resembles the most typical adult morphology (Olewnik, 2019).

These findings suggest that the proximal attachment of the FTM undergoes distinct morphogenetic phases during fetal development, and the observed heterogeneity may reflect either transient embryological states or the basis for adult variability. In particular, the high frequency of Type II and Type IV supports the hypothesis of a distal migration of the muscle origin with advancing gestational age, while Type III may represent either a developmental simplification or delayed myogenic differentiation (Bardeen and Lewis, 1901; Diogo et al., 2019).

Understanding these developmental variants provides critical insights into the phylogenetic plasticity of the anterior leg compartment musculature. Moreover, such morphogenetic variability may correlate with insertional types in later development and may have implications for the interpretation of congenital deformities, tendon harvesting, and imaging in pediatric orthopedics.

The classification proposed by Ruzik et al. (2022), based on 100 dissected fetal lower limbs (n = 50 fetuses; both sides included), distinguishes four morphologically distinct types of proximal tendon origin. These are summarized in Table 1.

**TABLE 1:** Fetal classification of the proximal attachment of the fibularis tertius (Ruzik et al., 2022).

Type	Description	Frequency (out of 100)
Type I	Origin from the upper third of the fibula and intermuscular septum	5 (5%)
Type II	Origin from the middle third of the fibula and intermuscular septum (most common)	21 (21%)
Type III	Fascial slip from extensor digitorum longus (EDL)	8 (8%)
Type IV	Origin from the distal third of the fibula and intermuscular septum	16 (16%)

### 2.2 Distal tendon classification in fetuses

The distal insertion of the FTM demonstrates considerable developmental variation during the fetal period. Notably, the muscle was present in only 50% of the dissected lower limbs, suggesting that the FTM is not universally expressed, even during late gestation. This aligns with postnatal prevalence data and supports the view of FTM as an evolutionarily emergent and developmentally variable structure.

Based on dissections of 100 fetal lower limbs (from 50 fetuses aged 18–38 weeks of gestation), Karauda et al. (2022) proposed a six-type classification system, which reveals distinct patterns of insertion onto lateral foot structures:

• Type I–Single distal attachment to the shaft of the fifth metatarsal; narrow, band-shaped morphology, observed in 9 limbs. This corresponds to adult Type I.
• Type II–Single, broad insertion into the base of the fifth metatarsal; fan-shaped morphology, found in 2 limbs. Equivalent to adult Type II.
• Type III–Single insertion into the shaft of the fourth metatarsal and adjacent fascia over the fourth interosseous space; observed in 6 cases. Considered a developmental precursor to adult Type V.
• Type IV–Insertion into the fascia of the fourth interosseous space, without direct bony attachment; seen in 10 cases. Interpreted as a transient or regressive embryological configuration.
• Type V–Bifurcated insertion: primary slip into the base of the fifth metatarsal and accessory slip into the base of the fourth; present in 7 cases. Morphologically equivalent to adult Type V.
• Type VI–Bifurcated insertion into the base of the fourth metatarsal and fascia of the fourth interosseous space; identified in 16 cases. Closely resembles adult Type VI, sometimes exhibiting fascial blending with the fibularis brevis tendon.

These findings suggest that complex distal variants including bifurcated and fascial insertions arise early in fetal development and may persist or evolve during postnatal maturation. Notably, fetal Types III–VI likely represent morphogenetic pathways leading to mechanically robust configurations adapted to bipedal gait.

Clinically, awareness of these fetal variants can aid in diagnosing congenital anomalies and predicting adult tendon architecture. Moreover, fascial expansions or accessory bands may have relevance for tendon grafting, ultrasound evaluation, or surgical decompression procedures in the lateral ankle region.

## 3 Morphological variability in adults

### 3.1 Proximal attachment types

The proximal attachment of the FTM in adults displays structured morphological variability, which was classified by Olewnik (2019) based on a cadaveric study of 106 adult lower limbs. Three distinct types of proximal origin were defined, primarily in relation to the fibular segment and fascial integration:

• Type 1 – The muscle originates from the distal half of the fibula and the anterior intermuscular septum. This was the most frequent configuration, observed in 67% of specimens. This form likely represents the standard anatomical pattern and provides a robust muscular belly with a long tendinous course.
• Type 2 – The origin is located more distally, on the distal third of the fibula and associated intermuscular septum. Present in 22% of cases, this type may exhibit a shorter muscle belly and a relatively longer tendon. It corresponds closely to some fetal configurations (particularly fetal Type IV), suggesting limited postnatal positional shift.
• Type 3 – The muscle lacks an independent belly and is represented by a fascial slip arising from the extensor digitorum longus. Found in 11% of adult limbs, this form reflects a simplified morphological pattern that closely mirrors fetal Type III, supporting the hypothesis of ontogenetic continuity from early developmental forms Ruzik et al. (2022) – Table 2.

**TABLE 2:** Classification of the proximal attachment of the fibularis tertius in adults Olewnik (2019).

Type	Description	Prevalence (n = 100)
Type 1	Origin from the distal half of the fibula with intermuscular septum involvement	67%
Type 2	Origin from the distal third of the fibula and associated septum	22%
Type 3	Fascial origin from the extensor digitorum longus muscle	11%

These findings suggest that the proximal morphology of the FTM in adults is not only variable but also developmentally conserved. The presence of a fascial slip (Type 3) is particularly notable as it challenges the assumption that the FTM always emerges as an independent muscle belly. Such variants may have important implications for surgical identification, tendon transfer planning, and ultrasound-based diagnostics, where thin or fascial slips may be overlooked (Pośnik et al., 2025; Pośnik et al., 2023).

Furthermore, correlation with fetal classifications (Ruzik et al., 2022) reinforces the notion that adult Types 1–3 are ontogenetically prefigured. Type 1 in adults corresponds to fetal Type II or IV, while adult Type 3 reflects an arrested or regressive pattern akin to fetal Type III.

### 3.2 Distal insertion types

Based on anatomical dissection of 106 adult lower limbs, Olewnik (2019) proposed a six-type classification of the distal attachment of the FTT, reflecting both structural diversity and potential clinical implications. The types differ in terms of insertion site, shape, and presence of accessory bands or fusion:

• Type I–Single distal attachment: the tendon inserts into the shaft of the fifth metatarsal bone. This was the most common variant, observed in 41 limbs (45%). It corresponds to a classical band-shaped morphology and represents the most mechanically efficient configuration for dorsolateral stabilization.
• Type II–Single distal attachment: the tendon exhibits a very wide insertion into the base of the fifth metatarsal bone. Seen in 20 limbs (22%), this variant offers greater contact area and may reflect increased functional demand. It aligns with fetal Type II and adult fan-shaped insertions.
• Type III–Single but complex insertion: the tendon inserts simultaneously into the base of the fifth metatarsal, the base and shaft of the fourth metatarsal, and the fascia covering the fourth interosseous space. This was identified in 15 limbs (16.5%), making it a morphologically expansive variant with probable embryological origins from bifurcated fetal insertions.
• Type IV–Bifurcated distal attachment: the main tendon inserts into the base of the fifth metatarsal, while an accessory band inserts into its shaft. This configuration was found in 8 limbs (8.8%) and suggests developmental partial duplication within a single metatarsal target.
• Type V–Bifurcated distal attachment: the main tendon inserts broadly into the base of the fifth metatarsal, while an accessory slip inserts into the base of the fourth metatarsal. This complex architecture was seen in 5 limbs (5.5%) and reflects developmental progression from fetal Type V.
• Type VI–Composite fusion: the FTT demonstrates fusion with an additional band from the fibularis brevis tendon, giving rise to the fourth dorsal interosseous muscle. This is the most structurally integrated variant, likely resulting from ontogenetic fascial blending. Although rare, its presence may significantly influence functional biomechanics and surgical dissection planes–Table 3.

**TABLE 3:** Classification of the distal attachment of the fibularis tertius in adults Olewnik (2019).

Type	Description	Prevalence (n = 91)
Type I	Insertion into the shaft of the fifth metatarsal	41 (45%)
Type II	Broad insertion into the base of the fifth metatarsal	20 (22%)
Type III	Insertion into base of fifth, fourth metatarsal, and fascia over 4th interosseous space	15 (16.5%)
Type IV	Bifurcated: base and shaft of the fifth metatarsal	8 (8.8%)
Type V	Bifurcated: base of fifth and fourth metatarsal	5 (5.5%)
Type VI	Fusion with fibularis brevis tendon; contributes to fourth dorsal interosseous muscle	2 (2.2%)

This classification reflects a spectrum from simple single-band insertions (Types I–II) to highly integrated and bifurcated arrangements (Types III–VI). Several types demonstrate clear embryological continuity with fetal morphotypes (Table 4) (Karauda et al., 2022), reinforcing the hypothesis that complex distal variants emerge early and are retained postnatally. Clinically, the recognition of such insertional diversity is critical during tendon transfer procedures, lateral foot surgeries, and imaging diagnostics, where accessory slips or fused structures may be misinterpreted as anomalies or pathological findings (Lee et al., 2013; Pośnik et al., 2025; Pośnik et al., 2023).

**TABLE 4:** Fetal classification of the distal attachment of the fibularis tertius Karauda et al. (2022).

Type	Description	Cases (n = 50)
Type I	Insertion into the shaft of the fifth metatarsal	9
Type II	Broad insertion into the base of the fifth metatarsal	2
Type III	Insertion into the shaft of the fourth metatarsal and fascia over the fourth interosseous space	6
Type IV	Insertion into the fascia over the fourth interosseous space	10
Type V	Bifurcated: base of the fifth and fourth metatarsal	7
Type VI	Bifurcated: base of the fourth metatarsal and fascia over the fourth interosseous space	16

### 3.3 Origin–insertion correlation

#### 3.3.1 Combined interpretation of proximal and distal variants

The morphological variability of the FTM encompasses both its origin and insertion, each exhibiting multiple distinct types in both fetal and adult populations (Karauda et al., 2022; Olewnik, 2019; Ruzik et al., 2022). Combined interpretation of these variants suggests a recurring anatomical association between specific proximal origins and corresponding distal insertions.

Adult Type 1 origins from the distal half of the fibula and intermuscular Septum were most commonly observed alongside Type I or II distal insertions, both of which represent simple, unifocal bony attachments to the fifth metatarsal. Similarly, Type 2 origins, which arise more distally from the fibula, were tended to appear with either Type II or Type IV insertions, suggesting that more caudal origin sites may give rise to broader or bifurcated tendon morphology.

Notably, Type 3 origins fascial slips from the extensor digitorum longus were disproportionately associated with Type V or VI insertions, both of which involve accessory bands or fascial fusion. This anatomical association suggests that reduced muscular differentiation proximally may be developmentally compensated by a wider or duplicated insertion footprint.

These observations imply that the architecture of the FTT is not independently determined at the proximal and distal poles, but rather develops as a continuum shaped by myogenic migration, tendon elongation, and fascial integration processes.

#### 3.3.2 Proposal of ontogenetic pathways from fetal to adult forms

An ontogenetic continuum can be proposed linking specific fetal and adult variants. For instance:

• Fetal Type II origin (middle third of fibula + septum) progresses to adult Type 1 origin (distal half + septum), while accompanying fetal Type II insertion (broad base of fifth metatarsal) persists as adult Type II.
• Fetal Type III origin (slip from extensor digitorum longus) correlates with adult Type 3 origin, both associated with distal insertions of Types V or VI.
• Fetal Type V insertion (bifurcated to 5th and 4th metatarsals) transitions into adult Type V, maintaining dual insertion but with increased complexity and wider fascial integration.

This integrated developmental perspective suggests potential predictive value of fetal tendon anatomy for postnatal morphology. It also supports the theory that some adult variants particularly Types III, V, and VI are not pathological or acquired, but rather represent conserved or elaborated embryonic morphologies (Diogo et al., 2019).

Clinically, this model enhances preoperative planning, particularly in pediatric surgery, tendon graft harvesting, or interpretation of variant anatomy in ultrasound and MRI. Ontogenetic classification may eventually inform new diagnostic frameworks based on structural lineage rather than isolated morphological endpoints.

To synthesize the observed morphogenetic continuity, Table 5 summarizes all known fetal and adult variants of FTM origin and insertion. This comparative framework highlights shared anatomical patterns and proposes developmental correlates between ontogenetic stages.

**TABLE 5:** Summary Comparison of Fibularis Tertius Attachment Types: Fetal vs. Adult (Based on Karauda et al., 2022; Olewnik, 2019).

Category	Type	Description	Prevalence	Adult equivalent
Fetal – Proximal	Type I	Upper third of fibula + intermuscular septum	5/50	Not directly matched
Fetal – Proximal	Type II	Middle third of fibula + intermuscular septum	21/50	Adult Type 1
Fetal – Proximal	Type III	Fascial slip from EDL	8/50	Adult Type 3
Fetal – Proximal	Type IV	Distal third of fibula + intermuscular septum	16/50	Adult Type 2
Fetal – Distal	Type I	Insertion into shaft of 5th metatarsal	9/50	Adult Type I
Fetal – Distal	Type II	Broad insertion into base of 5th metatarsal	2/50	Adult Type II
Fetal – Distal	Type III	Insertion to 4th metatarsal shaft + fascia	6/50	Adult Type V precursor
Fetal – Distal	Type IV	Fascia over 4th interosseous space	10/50	No direct adult counterpart
Fetal – Distal	Type V	Bifurcated to base of 5th and 4th metatarsal	7/50	Adult Type V
Fetal – Distal	Type VI	Bifurcated to 4th metatarsal + fascia	16/50	Adult Type VI
Adult – Proximal	Type 1	Distal half of fibula + septum	61/91	—
Adult – Proximal	Type 2	Distal third of fibula + septum	20/91	—
Adult – Proximal	Type 3	Fascial slip from EDL	10/91	—
Adult – Distal	Type I	Shaft of 5th metatarsal	41/91	—
Adult – Distal	Type II	Base of 5th metatarsal (broad)	20/91	—
Adult – Distal	Type III	Base of 5th + 6th + fascia	15/91	—
Adult – Distal	Type IV	Bifurcated: base + shaft of 5th	8/91	—
Adult – Distal	Type V	Bifurcated: base of 5th + 4th	5/91	—
Adult – Distal	Type VI	Fusion with fibularis brevis tendon	2/91	—

In order to further elucidate the developmental transition from fetal to adult configurations of the FTT, Table 6 presents a proposed ontogenetic progression model. This framework integrates anatomical observations with embryological interpretations, offering a mechanistic perspective on how individual tendon variants may evolve or regress during musculoskeletal maturation.

**TABLE 6:** Proposed ontogenetic progression of FTT attachment types.

Fetal type	Description	Proposed adult equivalent	Ontogenetic interpretation
Fetal Type I	Insertion into shaft of 5th metatarsal	Adult Type I	Stable linear development
Fetal Type II	Broad insertion into base of 5th metatarsal	Adult Type II	Expansion of insertion site during maturation
Fetal Type III	Insertion to 4th metatarsal + fascia	Adult Type V (precursor)	Bifurcation develops postnatally
Fetal Type IV	Fascia over 4th interosseous space	No direct adult equivalent	Regressive or transient form
Fetal Type V	Bifurcated to 5th and 4th metatarsal	Adult Type V	Early establishment of complex insertion
Fetal Type VI	Bifurcated to 4th metatarsal + fascia	Adult Type VI	Fusion and fascial integration

## 4 Topographic and fascial anatomy

### 4.1 Anatomical position of the FTM

The FTM is situated within the anterior compartment of the leg, lying lateral to the tibialis anterior and medial to the extensor digitorum longus (Olewnik, 2019; Standring, 2016). The FTM originates from the distal third of the fibula and the interosseous membrane, descending beneath both the superior and inferior extensor retinacula, often sharing a synovial sheath with the extensor hallucis longus tendon (Rourke et al., 2007). It inserts on the dorsum of the foot, typically on the fifth metatarsal, and is often palpable during resisted dorsiflexion or eversion, making it an accessible landmark in both physical examination and arthroscopic orientation (Taljanovic et al., 2015).

### 4.2 Anatomical relationship with the superficial fibular nerve

The superficial fibular nerve, a branch of the common fibular nerve (L4–S1), traverses the lateral compartment of the leg, initially deep to the fibularis longus and brevis muscles. It pierces the crural fascia typically in the distal third of the leg to become subcutaneous and continues anteriorly across the ankle joint (Standring, 2016; Yammine and Eric, 2017). In approximately 26% of individuals, the nerve follows an alternative path through the anterior compartment before piercing the fascia (Apaydin et al., 2008). Anatomically, it courses adjacent to the FTT, and variants involving a laterally displaced or accessory FTM belly may bring the tendon into close proximity with the superficial fibular nerve (Pośnik et al., 2025; Pośnik, Zielinska, Tubbs, Ruzik and Olewnik, 2023). This anatomical relationship carries clinical relevance during surgical interventions in the lateral compartment, fasciotomies, or tendon harvesting, where unrecognized nerve deviation or entrapment may result in iatrogenic injury (Karauda et al., 2022; Lee et al., 2013).

### 4.3 Fascial integration patterns and surgical implications

Fascial variation plays a central role in FTM anatomy, particularly in complex distal insertion types. In Type VI variants, as classified by Olewnik (2019), the FTT may partially or completely fuse with the fibularis brevis tendon or interosseous fascia, forming a structural continuum that may contribute to the formation of the fourth dorsal interosseous muscle. These fascial networks can obscure standard dissection planes and alter the topography of adjacent neurovascular structures. Clinically, this is especially important during procedures such as peroneal tendon transfers or decompression surgeries, where failure to recognize blended fascial anatomy may lead to incomplete tendon release or inadvertent damage to neighboring nerves (Camacho et al., 2019; Iceman et al., 2020).

### 4.4 Imaging considerations

Variant FTT and fascial blends may present diagnostic challenges on musculoskeletal imaging. On ultrasound and MRI, accessory bands or fused tendons of the FTM may be misinterpreted as pathological structures such as fibrotic strands, scar tissue, or partial tendon tears (Lee et al., 2013; Taljanovic et al., 2015). In these cases, correlation with topographic anatomy and dynamic ultrasound assessment can improve diagnostic accuracy and prevent unnecessary intervention.

## 5 Comparative anatomy across mammals–phylogenetic perspective

### 5.1 Carnivores (Canidae, Felidae)

In carnivorous mammals such as Canidae (e.g., *Canis lupus familiaris*) and Felidae (e.g., *Felis catus*), the FTM is consistently absent. Anatomical dissections have confirmed that these species lack any muscle equivalent to the human FTT that inserts onto the fifth metatarsal (Diogo and Abdala, 2010b; Heilmann, 1926). Instead, the extensor digitorum longus emerges as the principal dorsiflexor of the paw and is supported by a simplified lateral muscle group composed mainly of fibularis longus and brevis (Diogo, 2007; Diogo and Abdala, 2007).

This muscular architecture reflects functional specialization toward digitigrade locomotion, in which rigid leverage from the tarsus to the digits is prioritized over independent eversion or selective dorsiflexion of the lateral rays. The evolutionary absence of FTM in these taxa is therefore interpreted as a phylogenetically stable trait, shaped by biomechanical efficiency rather than neuromuscular versatility (Abdala and Diogo, 2010; Diogo, 2005).

Furthermore, the absence of accessory fibular muscles or extensor digitorum longus slips to the fifth metatarsal in these carnivores distinguishes them from primate or ungulate configurations and supports the hypothesis that the FTM evolved in association with upright locomotion and fine motor control of the lateral forefoot.

### 5.2 Rodents and lagomorphs

In Rodentia (e.g., *Rattus norvegicus*, *Mus musculus*) and Lagomorpha (e.g., *Oryctolagus cuniculus*), no distinct FTM is observed. The muscular composition of the lateral and anterior compartments of the leg is simplified compared to primates and large cursorial mammals. Although small fibular or extensor slips directed toward the lateral metatarsals may be present, they do not form an anatomically or functionally independent muscle belly and lack separate neurovascular supply (Diogo, 2007; Diogo and Abdala, 2007; Diogo and Abdala, 2010a; Diogo and Abdala, 2010b).

These rudimentary slips typically arise from the extensor digitorum longus or fibularis brevis and may insert into the fascia or tendons near the fifth digit, but their presence is inconsistent and developmentally variable. Their embryological identity remains ambiguous, and they are generally interpreted as remnants of primitive lateral leg musculature rather than true homologues of the human FTM (Diogo et al., 2009a; Diogo et al., 2009b).

Functionally, rodents and lagomorphs exhibit plantigrade or semi-digitigrade locomotion with minimal demand for isolated lateral toe dorsiflexion, which likely explains the evolutionary regression of the FTM in these taxa (Abdala and Diogo, 2010; Diogo and Abdala, 2010a).

### 5.3 Chiroptera

In Chiroptera (bats), the anatomy of the hindlimb diverges markedly from that of other eutherian mammals due to the demands of flight and roosting. The FTM is absent in this order. Instead, the musculature of the distal hindlimb is highly modified and reduced, reflecting a decreased need for terrestrial locomotor specialization (Abdala and Diogo, 2010; Diogo, 2007; Diogo and Abdala, 2007; Diogo and Abdala, 2010a; Diogo and Abdala, 2010b).

Bats rely predominantly on their forelimbs for flight, while the hindlimbs function primarily in clinging, hanging, or limited crawling. The extensor digitorum longus is often present but diminished in size and force compared to cursorial mammals, and no distinct dorsiflexor comparable to the FTM has been identified in comparative anatomical studies of chiropteran species (Abdala and Diogo, 2010; Diogo, 2010; Diogo and Abdala, 2010a).

Moreover, the configuration of the ankle joint in bats is adapted to static suspension, including locking mechanisms and altered tendon routing, which negates the biomechanical need for a muscle inserting into the fifth metatarsal for lateral dorsiflexion (Diogo et al., 2009a; Diogo et al., 2009b; Heilmann, 1926). The evolutionary regression of dorsolateral foot muscles in Chiroptera further underscores the FTM’s association with weight-bearing and terrestrial bipedal or quadrupedal locomotion.

### 5.4 Marsupials

In marsupials (e.g., *Macropus* spp., *Didelphis virginiana*), the FTM is consistently absent. Dissections and developmental analyses reveal no true FTT inserting into the fifth metatarsal. However, in some specimens, rudimentary slips arising from the extensor digitorum longus or adjacent fascia have been documented (Diogo, 2007; Diogo and Abdala, 2007; Diogo and Abdala, 2010a; Diogo and Abdala, 2010b). These slips may extend toward the lateral metatarsals or digits, but they lack independent morphology or innervation and are not homologous to the human FTM.

From a functional standpoint, marsupial locomotion ranging from saltatory (e.g., kangaroos) to scansorial (e.g., opossums) relies more heavily on proximal musculature and axial stability than on fine distal dorsiflexion. The absence of an FTM analogue reflects both phylogenetic constraint and a locomotor strategy that minimizes the functional necessity of a specialized lateral tendon inserting into the fifth metatarsal (Abdala and Diogo, 2010).

Embryological data suggest that the developmental precursors of the FTM may regress or be incorporated into other muscle groups during morphogenesis in marsupials, echoing a broader evolutionary trend of simplification in distal limb musculature compared to placental mammals (Diogo et al., 2009a; Diogo et al., 2009b).

### 5.5 Ungulates (Artiodactyla, Perissodactyla)

In ungulates, encompassing both Artiodactyla (e.g., *Bos taurus*, *Sus scrofa*) and Perissodactyla (e.g., *Equus caballus*, *Tapirus terrestris*), the FTM is consistently absent. These taxa exhibit a marked shift toward digitigrade or unguligrade locomotion, wherein the mechanical role of foot dorsiflexion is dominated by robust digital extensor muscles such as the extensor digitorum longus and extensor digitorum lateralis (Abdala and Diogo, 2010; Diogo and Abdala, 2010a; Diogo and Abdala, 2010b; Heilmann, 1926).

In perissodactyls such as the horse, a well-developed muscle historically referred to as “peroneus tertius” is present but functionally and anatomically distinct from the human FTM. It originates from the extensor fossa of the femur and inserts broadly on the tarsus and metatarsus, acting as a mechanical linkage in the reciprocal apparatus rather than as an independent dorsiflexor of the fifth metatarsal (Abdala and Diogo, 2010; Diogo, 2010; Diogo and Abdala, 2010a). This structure, although eponymously named, is not homologous to the FTM and lacks association with the anterior compartment or distal fibula.

In artiodactyls (e.g., cattle, pigs), no such analog is found; the fibularis muscle group is reduced, and tarsal extension is largely achieved by the gastrocnemius and digital extensors. Embryological studies suggest that any FTM-related mesenchyme is either lost or integrated into broader extensor complexes during development (Diogo et al., 2009a; Diogo et al., 2009b).

Thus, in ungulates, the evolutionary absence or deep transformation of FTM correlates with specialization for cursorial locomotion and passive stabilization of the distal limb.

### 5.6 Aquatic mammals (Cetacea, Sirenia)

In fully aquatic mammals such as Cetacea (e.g., dolphins, whales) and Sirenia (e.g., manatees, dugongs), the hindlimb musculature is profoundly regressed or entirely absent. During embryonic development, transient limb buds appear but subsequently regress, resulting in vestigial or completely lost pelvic limbs in adults (Diogo, 2010; Diogo and Abdala, 2010a; Diogo et al., 2009a; Diogo et al., 2009b).

No structure homologous to the FTM is retained in these taxa. Indeed, the entire posterior limb apparatus including bones, joints, and muscles is either reduced to non-functional remnants (as in some odontocetes) or completely lost (as in mysticetes) (Abdala and Diogo, 2010; Diogo, 2010; Diogo and Abdala, 2010a). The loss of the FTM is thus a secondary evolutionary reduction linked to the transition from terrestrial quadrupedalism to aquatic locomotion.

In manatees and dugongs (Sirenia), although rudimentary pelvic bones persist, no functional hindlimb musculature is present in adult specimens. The absence of dorsiflexor muscles, including FTM or its equivalents, reflects complete biomechanical reliance on axial musculature and caudal propulsion for movement.

These observations reinforce the notion that the FTM is a locomotor-dependent muscle, whose retention and development correlate with weight-bearing and terrestrial propulsion demands both absent in aquatic environments.

### 5.7 Primates

#### 5.7.1 Homo sapiens

In modern humans, the FTM is variably present, with reported prevalence ranging from 38% to nearly 100% depending on the population and methodology (Olewnik, 2019; Pośnik et al., 2025; Yammine and Eric, 2017). It arises from the distal fibula or extensor digitorum longus, traverses the anterior compartment of the leg, and inserts into the fifth metatarsal typically its shaft or base.

The FTM is functionally linked to bipedalism, contributing to lateral foot stabilization during the stance phase and assisting in eversion and dorsiflexion. Its development and persistence in humans are thought to reflect adaptation to upright locomotion, where fine control of mediolateral foot balance is essential (Jana and Roy, 2011; Jungers et al., 1993).

Importantly, the FTM shows significant morphological variability, both proximally and distally. Adult classifications (Types I–VI) capture this range of insertions from simple shaft insertions to bifurcated tendons and fascial fusion with fibularis brevis (Olewnik, 2019). Ontogenetic studies reveal that this variation arises prenatally, supporting the hypothesis that morphogenesis of the FT is developmentally regulated and potentially influenced by mechanical factors (Karauda et al., 2022; Ruzik et al., 2022).

From a clinical perspective, the FTM has been implicated in lateral ankle pathology, fibular tendon syndrome, and iatrogenic injury during anterior ankle procedures. Its tendon may also be misinterpreted as pathological on ultrasound or MRI when presenting in atypical forms (Bencardino and Rosenberg, 2001; Iceman et al., 2020; Pośnik et al., 2023).

Thus, in *Homo sapiens*, the FTM represents a functionally significant but evolutionarily young structure, whose variation carries anatomical, diagnostic, and surgical relevance.

#### 5.7.2 *Pan* (chimpanzee)

In chimpanzees (*Pan troglodytes*), the FTM is typically absent or rudimentary, with only rare instances of identifiable slips arising from the extensor digitorum longus or distal fibula (Diogo and Abdala, 2010a; Diogo and Abdala, 2010b; Heilmann, 1926). When present, such slips do not form a fully differentiated muscle belly and generally lack the distinct insertion into the fifth metatarsal seen in humans.

The absence or underdevelopment of the FTM in *Pan* is consistent with its partially arboreal and knuckle-walking locomotion, in which lateral foot stabilization and precise dorsiflexion of the fifth ray are not essential. Unlike *Homo sapiens*, chimpanzees maintain grasping abilities in the foot and show minimal specialization for sustained bipedalism, reducing the evolutionary pressure for structures like the FTM (Diogo et al., 2009a; Diogo et al., 2009b; Jungers et al., 1993).

Developmentally, the FTM in chimpanzees may correspond to regressive or incomplete expression of ancestral mesenchyme, possibly reflecting a transitional phylogenetic state. This aligns with the broader pattern observed in great apes, where muscle variability in the distal leg is more pronounced than in humans, but less functionally constrained (Abdala and Diogo, 2010; Diogo, 2010; Diogo and Abdala, 2010a).

Thus, in *Pan*, the FTM can be considered vestigial at best, with partial terrestriality insufficient to drive consistent evolutionary retention.

#### 5.7.3 *Gorilla*, *Pongo*

In both gorillas (*Gorilla gorilla*) and orangutans (*Pongo pygmaeus*), the FTM is consistently absent. Extensive anatomical surveys confirm the lack of a distinct muscle originating from the distal fibula and inserting onto the fifth metatarsal in these species (Abdala and Diogo, 2010; Diogo and Abdala, 2010a; Diogo and Abdala, 2010b; Diogo et al., 2010; Heilmann, 1926). No comparable muscular structure with a similar topography or function has been documented.

This absence is attributed to their strongly arboreal adaptations, particularly in *Pongo*, where foot use emphasizes prehensile function over locomotor stability. Both genera rely heavily on plantigrade stance, vertical climbing, and grasp mechanics, which demand powerful flexors and intrinsic foot muscles, rather than lateral stabilizers like the FTM (Diogo and Abdala, 2010a; Diogo and Abdala, 2010b; Diogo et al., 2009a; Diogo et al., 2010; Diogo et al. 2009b). Even in *Gorilla*, where terrestrial knuckle-walking is prominent, the foot remains oriented toward weight-bearing via the medial longitudinal arch, not eversion or lateral stabilization.

From an evolutionary perspective, the FTM appears as a derived structure in humans, associated with sustained bipedal locomotion, and thus is not retained in non-human hominids that did not undergo similar postural and functional shifts (Jungers et al., 1993). This is consistent with broader musculoskeletal simplification in the distal limb of arboreal apes, where muscle complexity is conserved primarily in the digits and proximal flexors (Abdala and Diogo, 2010).

#### 5.7.4 Cercopithecoids

In cercopithecoid primates (e.g., *Macaca mulatta*, *Papio anubis*), the presence of a distinct FTM is generally absent, though FTM-like muscular slips can occasionally be observed. These slips typically arise from the extensor digitorum longus or distal fibula and extend toward the fifth or fourth metatarsal, but they do not form a fully isolated muscle belly and lack independent innervation (Abdala and Diogo, 2010; Diogo and Abdala, 2010a; Diogo and Abdala, 2010b; Heilmann, 1926).

Such structures may represent transitional or rudimentary homologues, yet their variable presentation across species and specimens suggests functional redundancy rather than evolutionary refinement. In *Macaca* and *Papio*, the hindlimb is well-adapted to both quadrupedalism and arboreal leaping, favoring generalized extensor systems over specialized lateral stabilizers like FTM (Abdala and Diogo, 2010; Diogo et al., 2009a; Diogo et al., 2009b).

Functionally, these primates rely on digital flexors and long extensors for foot positioning and propulsion. The relative absence of FTM reflects low demand for isolated fifth ray dorsiflexion, and the persistence of only non-isolated slipssupports the notion that the FTM is a derived specialization unique to hominins (Jungers et al., 1993).

Embryological evidence from comparative developmental studies confirms that while limb bud musculature in cercopithecoids includes fibular components, they often undergo differential fusion or regression in later stages (Abdala and Diogo, 2010; Diogo, 2010; Diogo and Abdala, 2010a).

#### 5.7.5 Tarsiers and strepsirrhines

In tarsiers (*Tarsius* spp.) and strepsirrhines (e.g., *Lemur*, *Loris*, *Galago*), the FTM is entirely absent. Detailed anatomical and myological investigations confirm that no muscle originates from the distal fibula and inserts onto the fifth metatarsal with the topographic or functional properties of the human FT (Diogo and Abdala, 2010a; Diogo and Abdala, 2010b; Heilmann, 1926). Even minor fibular slips or extensor digitorum longus offshoots directed laterally are not observed in these basal primates.

This absence reflects a highly conserved arboreal adaptation, in which the hindfoot is optimized for grasping and leaping, not for precise lateral stabilization or dorsiflexion of the fifth ray. The calcaneus and navicular complex, along with strong intrinsic plantar flexors, dominate foot function in these taxa, rendering structures like FTM functionally unnecessary (Diogo, 2010; Diogo and Abdala, 2010a; Diogo et al., 2009a; Diogo et al., 2009b).

From a phylogenetic standpoint, tarsiers and strepsirrhines represent early-diverging primate lineages that retain many ancestral features, including generalized distal limb musculature. Their locomotion emphasizes grasp-force transmission and vertical clinging, with minimal evolutionary pressure toward the development of a lateral dorsiflexor like FTM (Abdala and Diogo, 2010).

As such, the complete absence of FTM in these groups strengthens the hypothesis that the muscle is a derived hominin feature, linked closely to bipedalism and terrestrial loading.

A cross-taxonomic comparison of FTMpresence among mammals is summarized in Table 7. This phylogenetic analysis underscores the evolutionary novelty of FTM in humans and its functional dissociation from the extensor digitorum longus in other taxa.

**TABLE 7:** Comparative presence of the fibularis tertius muscle across mammalian taxa.

Taxonomic group	Presence of FT	Remarks and citations
Carnivores (e.g., Canidae, Felidae)	Absent	EDL is the dominant dorsiflexor; no FT analog present Heilmann (1926)
Rodents/Lagomorphs	Absent or rudimentary slips	Typically EDL extensions without independent muscular belly Diogo et al. (2009a)
Chiroptera (bats)	Absent	No discrete dorsiflexor homologs reported; limb adapted for flight Diogo and Abdala (2010b)
Marsupials (e.g., kangaroos)	Absent	Occasional EDL slips reported; no true FT analogue Diogo et al. (2010)
Ungulates (e.g., horses, pigs)	Absent	Digital extensors dominate; lateral dorsiflexors largely lost Diogo et al. (2009b)
Aquatic mammals (Cetacea, Sirenia)	Absent	Hindlimbs are vestigial or lost; no FT present Diogo and Wood (2012)
Cercopithecoid monkeys	Variable or rudimentary	Non-isolated FT-like slips occasionally noted near EDL Diogo et al. (2009b)
Great apes (Gorilla, Pongo)	Absent	No reports of FT; limb adapted for arboreal grasping Diogo and Wood (2012)
Chimpanzees (Pan)	Rudimentary or absent	Occasional fascial separations from EDL reported; no independent function Diogo et al. (2009b)
Humans (*Homo sapiens*)	Present (variable)	Prevalence ranges from 30% to 90%; linked to bipedalism and lateral stabilization Yammine and Eric (2017)

### 5.8 Evolutionary and developmental implications

#### 5.8.1 FT as an evolutionary novelty

The FTM stands out as a genuinely novel evolutionary structure in the human lower limb musculature. Early anatomical literature often mischaracterized it as vestigial, implying regression from a more functionally important ancestral muscle (Anson and McVay, 1971; Macalister, 1875). However, systematic comparative analyses across mammals including non-human primates demonstrate that the FTM is largely absent in quadrupeds, arboreal species, and even terrestrial great apes (Diogo and Abdala, 2010a; Diogo and Abdala, 2010b; Heilmann, 1926). This absence suggests that the FTM is not a retained ancestral muscle, but rather an evolutionary innovation that emerged within the hominin lineage, uniquely adapted to the demands of sustained bipedalism (Jungers et al., 1993; Yammine and Eric, 2017).

Unlike conserved limb muscles with stable homologies across taxa, the FTM is inconsistent even within human populations, further supporting its evolutionary recentness and developmental plasticity (Olewnik, 2019; Ruzik et al., 2022). Its phylogenetic emergence appears correlated with a functional shift from generalized arboreality to habitual terrestrial bipedality a major transition in hominin evolution.

#### 5.8.2 Biomechanical rationale for FT emergence

The development of the FTM can be understood in light of the biomechanical transformations accompanying upright gait. Bipedal locomotion imposes distinct mechanical stresses on the lower limb, particularly during single-limb stance, when the body weight must be balanced over a narrow base of support. This configuration demands precise control of eversion and dorsiflexion at the lateral aspect of the foot (Jana and Roy, 2011; Jungers et al., 1993).

In this context, the FTM serves to stabilize the lateral longitudinal arch, assist the extensor digitorum longus in toe clearance, and reduce inversion torque during push-off. Its action complements the tibialis anterior and fibularis longus, contributing to dynamic foot positioning during both swing and stance phases of gait. This supports the hypothesis that the FTM evolved as a functional solution to balance and lateral propulsion challenges imposed by bipedalism demands that are absent or diminished in quadrupeds and climbers.

#### 5.8.3 Developmental origin and myogenic differentiation

From a developmental perspective, the FTM appears to derive from lateral extensions or fascial differentiations of the extensor digitorum longus muscle mass. Embryological studies indicate that in early limb bud formation, the muscle anlagen of extensor digitorum longus and FTM are not separately demarcated. Instead, the FTM emerges as a lateral fascial condensation or split, gradually acquiring its own tendon and, in many cases, partial fascial compartmentalization (Karauda et al., 2022; Ruzik et al., 2022).

This process mirrors the “splitting hypothesis” of muscle evolution, whereby novel muscles evolve through partial duplication or differentiation of existing myogenic fields (Diogo, 2007; Diogo and Abdala, 2007; Diogo and Abdala, 2010a; Diogo and Abdala, 2010b; Diogo et al., 2009a). Importantly, the FTM does not arise from a unique blastema, but rather from a modification of pre-existing mesodermal derivatives, most likely driven by altered mechanical loading and neural patterning in response to erect posturę (Bardeen, 1906; Bardeen and Lewis, 1901; Diogo et al., 2009a; Diogo et al., 2009b).

The variability of FTM in fetal cadaver studies further supports this notion. Karauda et al. (2022) and Ruzik et al. (2022) demonstrated that both the origin and insertion patterns of the FTM are established prenatally, yet show a wide spectrum of configurations. This suggests that genetic and epigenetic influences, including positional information and mechanical stimulation, guide its partial differentiation.

#### 5.8.4 Broader evolutionary significance

The FTM may serve as a model for understanding how new muscles arise during macroevolutionary transitions. Its emergence illustrates a typical scenario in vertebrate musculoskeletal evolution: functional pressure here, from bipedal gait leads to regional specialization, followed by myogenic and neural divergence, and finally, morphological fixation in the adult phenotype. This process aligns with Diogo’s framework for muscle evolution involving convergent and divergent modifications of conserved topologies (Diogo, 2010; Diogo and Abdala, 2010a; Diogo and Wood, 2012).

Furthermore, the FTM exemplifies the evolution–development interface, in which ontogeny recapitulates selective pressures encountered during phylogeny. Unlike true atavistic or degenerate structures, the FTM exhibits a progressive, lineage-specific emergence, which reinforces its classification as a derived adaptation, not a relic of past morphologies.

A phylogenetic overview of the presence and functional relevance of the FTM across mammalian clades is summarized in Table 8.

**TABLE 8:** Summarizes the presence or absence of the fibularis tertius (FT) muscle across major mammalian clades, alongside the dominant functional adaptations of the hindlimb. The data highlight the evolutionary novelty of the FT in *Homo sapiens* and its absence or rudimentary analogues in non-human primates and other mammals.

Clade/Species	Presence of FT	Functional role
Cetacea	Absent	N/A
Sirenia	Absent	N/A
Artiodactyla	Absent	Digital extension
Perissodactyla	Absent	Reciprocal apparatus
Carnivora	Absent	EDL dominant
Rodentia/Lagomorpha	Absent (rudimentary slips possible)	Dorsiflexors generalized
Chiroptera	Absent	Wing support
Marsupialia	Rare EDL slips	Diverse, arboreal
Strepsirrhines	Absent	Grasping
Tarsiers	Absent	Grasping
Cercopithecoidea	FT-like slips (non-isolated)	Quadrupedal locomotion
Pongo	Absent	Knuckle-walking
Gorilla	Absent	Knuckle-walking
Pan	Rudimentary/Absent	Partial terrestriality
*Homo sapiens*	Present (variable)	Bipedalism, lateral foot stabilization

## 6 Functional and biomechanical role

### 6.1 Contribution to eversion, dorsiflexion, and lateral stabilization

The FTM plays a unique and nuanced role in foot kinematics, contributing significantly to eversion and dorsiflexion of the foot, particularly during the swing phase of gait. Its tendon inserts onto the dorsal aspect of the fifth metatarsal, allowing it to act in concert with the extensor digitorum longus and tibialis anterior to elevate and stabilize the lateral column of the foot (Jana and Roy, 2011; Jungers et al., 1993).

During the initial swing phase, the FTM assists in toe clearance by contributing to dorsiflexion, especially when extor digitorum longus activation alone is insufficient for lateral foot control. In the terminal swing, its role in controlling inversion becomes biomechanically relevant, as the foot prepares for heel strike. The presence of FTM has been associated with enhanced resistance to inversion sprains, particularly in individuals engaged in repetitive or high-impact locomotion (Yammine and Eric, 2017).

Biomechanical models and EMG studies suggest that FTM activation patterns mirror those of extensor digitorum longus, yet show distinct bursts during lateral load shift, indicating a proprioceptive and stabilizing function not shared by the other anterior compartment muscles (Camacho et al., 2019). This is especially critical in bipedal gait, where balance must be maintained over a narrow support base.

From a functional morphology perspective, the FTM complements the fibularis longus and brevis in providing dynamic lateral stability, but unlike tchem acts as a dorsiflexor rather than plantarflexor, making it unique in both mechanical axis and moment arm. Its absence may be compensated by synergists, but this often comes at the cost of reduced lateral control, particularly during rapid directional changes.

In summary, the FTM serves as a minor but strategically placed muscle, optimized for fine-tuned foot positioning, dynamic balance, and injury prevention in bipedal locomotion.

### 6.2 Fine-tuning of foot trajectory in bipedal gait and dynamic movements

The FTM is uniquely positioned to assist in real-time modulation of foot trajectory, particularly during bipedal gait and rapid directional changes. The FTT insertion on the fifth metatarsal allows for a lateral vector of forcethat contributes not only to eversion, but also to subtle adjustments in foot orientation required for balance and responsiveness on uneven or shifting terrain (Jana and Roy, 2011; Jungers et al., 1993).

In activities that demand quick acceleration, deceleration, or directional transitions such as running, cutting, or pivoting the FTM provides dynamic lateral tension, which fine-tunes the position of the lateral forefoot. This role is especially relevant during the terminal swing and pre-stance phases, where preparatory positioning of the foot is critical for safe landing and propulsion (Camacho et al., 2019).

Additionally, the FTM may function synergistically with the tibialis anterior and fibularis muscles to enhance postural control and mitigate excessive inversion moments that contribute to ankle sprains. Its presence is associated with reduced incidence of lateral instability, particularly in athletes and individuals undergoing high-frequency changes in locomotor vector (Lee et al., 2013; Yammine and Eric, 2017).

Though often small and sometimes absent, the FTM reflects a highly specialized adaptation that augments precision neuromuscular control, distinguishing human lower limb mechanics from those of quadrupedal primates and arboreal mammals.

### 6.3 Influence of insertional variants on mechanical Performance

The mechanical function of the FTM is closely modulated by its insertional morphology, which exhibits considerable interindividual variability. As demonstrated by Olewnik (2019), FTT insertions can range from simple linear insertions on the shaft of the fifth metatarsal (Type I) to more complex bifurcated or fan-shaped insertions (Types IV–VI). These anatomical variants have direct implications for force transmission, moment arm configuration, and functional efficacy during gait and dynamic loading.

For instance, Type III variants, which include wide insertions onto the base and shaft of the fifth metatarsal and sometimes extend to the sixth ray or adjacent fascia, may distribute force across multiple axes, thereby diminishing the net dorsiflexion or eversion torque generated during activation. This could theoretically reduce the biomechanical efficiency of the muscle, particularly in tasks requiring precision or rapid eversion (Olewnik, 2019; Yammine and Eric, 2017).

In contrast, fan-shaped insertions (e.g., Type V), which involve a broad area of tendon contact or bifurcation into multiple metatarsals, may increase torque potential by enhancing the muscle’s lever arm and distributing tensile load over a wider surface. These configurations could potentially offer greater lateral stabilization, especially during high-velocity foot motions, and may have protective effects against ankle inversion.

Importantly, the fusion-type variant (Type VI), in which FTT merges with the fibularis brevis tendon, likely alters its independent function entirely transforming it from a discrete dorsiflexor to a shared muscular structure involved in more complex eversion mechanics. Such a variant may result in redundancy or loss of specific vector control, with unclear implications for dynamic foot stabilization.

Recognizing these morphological types is essential in both diagnostic imaging and surgical planning, especially during tendon harvesting or lateral ankle reconstruction, where variant anatomy can influence graft choice and functional outcomes.

## 7 Radiological features and diagnostic challenges

### 7.1 Under-recognition in MRI and ultrasound

Due to its relatively small size, anatomical variability, and proximity to the extensor digitorum longus, the FTM can be difficult to identify in routine radiologic studies. On MRI, especially in standard axial planes, the FTT often blends with the extensor digitorum longus belly or tendon, particularly in individuals with Type III or VI variants. This anatomical blending may lead to under-reporting or complete omission of the FTT in imaging interpretations (Lee et al., 2013; Taljanovic et al., 2015).

Ultrasound identification of the FTT requires high-resolution probes and a systematic scanning protocol along the anterior aspect of the fibula, following the tendon distally towards its insertion. In many cases, the FTT is overlooked because of its thin caliber and the operator’s insufficient awareness of its variant anatomy (Bianchi et al., 2010; Bianchi et al., 2007). Its subretinacular course and adjacency to the EDL compound these difficulties, particularly in fan-shaped or bifurcated variants that may alter its imaging signature.

### 7.2 Mimicry of pathologies

Morphological variants of the FTT, particularly those with wide insertions or fusion patterns (e.g., Type VI), may mimic a range of pathological entities. These include longitudinal tendon tears, bifid tendons, peroneus quartus or digiti quinti anomalies, synovial sheath expansions, or atypical ganglion cysts (Walls et al., 2023; Yammine, 2015). On MRI, such variants may present as thickened or duplicated structures in the lateral retromalleolar region, leading to misdiagnoses. Fusion of FTT with the fibularis brevis may create the appearance of anomalous or accessory musculature, when in fact these are benign anatomical variants.

Additionally, the FTT may coexist with intratendinous ganglion cysts, fascial expansions, or accessory muscular slips, further complicating radiologic interpretation (Chaney et al., 2017; Sookur et al., 2008). These findings are particularly misleading in patients with lateral ankle pain or instability, where such variants can be mistaken for degenerative lesions or soft-tissue masses.

### 7.3 Morphological variants as diagnostic pitfalls

Types IV to VI of the FTT display complex insertion patterns that may result in significant diagnostic pitfalls. On coronal and sagittal MRI planes, fan-shaped insertions or bifurcated tendons may simulate partial tendon ruptures or soft-tissue neoplasms. In patients with FTT agenesis, hypertrophy or course deviation of adjacent muscles (extensor digitorum longus, fibularis brevis) can falsely suggest tendinopathy or anomalous course (Bencardino and Rosenberg, 2001; Taljanovic et al., 2015).

These challenges necessitate a multiphasic and multiplanar imaging approach. Radiologists must be familiar with the full spectrum of FTT variants to avoid false positives and to provide accurate preoperative mapping. Dedicated protocols for lateral ankle and dorsal foot imaging should routinely consider the FTT, particularly in contexts of trauma, eversion instability, or post-surgical evaluation.

## 8 Clinical and surgical implications

### 8.1 Tendon harvesting and graft planning

Detailed knowledge of the FTT’s anatomical variability is critical in tendon harvesting and anterior compartment surgical procedures. Although the FTT is not a primary donor tendon, it may be encountered incidentally during tendon graft harvest or exposure for anterior ankle stabilization, reconstruction, or tendon transfer surgeries. Complex or fan-shaped insertions (e.g., Type V or VI) can result in intraoperative confusion and unintended injury to adjacent structures. Moreover, fusion with the fibularis brevis may complicate identification of individual tendons (Olewnik, 2019; Yammine and Eric, 2017). Preoperative imaging and intraoperative recognition of FTT variants improve surgical precision and reduce operative time.

### 8.2 Biomechanical implications and lateral ankle instability

FTT agenesis, present in approximately 10%–15% of individuals, may contribute to functional lateral column insufficiency. The FTT supports dorsiflexion and eversion, particularly during the swing phase, providing additional control over foot clearance and balance. Its absence may shift mechanical load to the fibularis brevis and longus, predisposing to peroneal overuse syndromes or Jones fractures (Iceman et al., 2020; Yammine and Eric, 2017). Clinically, FTT presence may serve as a protective factor against inversion sprains (Camacho et al., 2019), while its absence could be included in risk stratification for lateral instability, especially in athletes and individuals with high mechanical demands on the lateral foot.

### 8.3 Neurological and functional assessment

As the FTT is innervated by the superficial fibular nerve, its function can serve as a clinical marker of nerve integrity. Manual muscle testing (resisted dorsiflexion + eversion) may detect subtle neuropathies or compression syndromes affecting this nerve branch. In post-traumatic or post-surgical contexts, FTT dysfunction may indicate localized injury or neuropraxia, particularly after fasciotomy or ankle arthroscopy (Apaydin et al., 2008). EMG studies support the utility of FTT activation analysis in assessing superficial fibular nerve function and proprioceptive integrity (Jana and Roy, 2011). These findings are especially relevant in neuro-orthopaedic and post-reconstructive care.

### 8.4 Variant morphology in surgical navigation

Fusion variants of FTT (e.g., Type VI) present challenges in dissection and surgical navigation. These configurations may cause misidentification of tendon borders, overlap with the fibularis brevis, or shift expected topography of neurovascular elements. In surgeries addressing lateral ankle reconstruction, tendon transfers, or even external compartment decompressions, unrecognized FTT morphology may increase the risk of iatrogenic injury or result in incomplete repair (Olewnik, 2019; Walls et al., 2023). Advanced preoperative planning, anatomical familiarity, and use of magnification are advisable. Incorporating FTT variant awareness into resident education and surgical anatomy curricula may reduce intraoperative error and improve long-term outcomes.

A structured overview of the surgical and radiological considerations associated with each classified distal variant of the FTT is presented in Table 9. The table aims to support preoperative planning, differential diagnosis, and radiological interpretation in cases involving lateral foot pain or tendon anomalies.

**TABLE 9:** Clinical and imaging implications of fibularis tertius variants.

Variant type	Anatomical description	Clinical implications	Imaging considerations
Type I	Narrow, single insertion into shaft of 5th metatarsal	Standard morphology; minimal surgical risk	May be inconspicuous on US/MRI; unlikely to mimic pathology
Type II	Broad, fan-like insertion into base of 5th metatarsal	Potentially increased mechanical leverage; relevant for tendon harvesting	Clearly seen on MRI; may resemble hypertrophic tendon or anomaly
Type III	Insertion to 5th and 6th metatarsals with fascial slip	Possible cause of dorsal foot pain or lateral compartment impingement	May present as bifid or irregular tendon structure; requires careful interpretation
Type IV	Split insertion to shaft and base of 5th metatarsal	Relevant in trauma cases and surgical planning; may contribute to traction stress	Can mimic split tendon tear; high-resolution imaging beneficial
Type V	Dual insertion to base of 5th and 4th metatarsals	Risk of altered load transmission; association with metatarsalgia	May appear as anomalous or accessory structure; confirmed by comparative side imaging
Type VI	Fusion with fibularis brevis and fascia	Surgical dissection may risk nerve entrapment; contraindicated for graft	Complex appearance; possible misinterpretation as mass or hypertrophy on US/MRI

## 9 Future directions

Despite increasing anatomical and clinical interest in the FTM, multiple research avenues remain unexplored. The unique combination of phylogenetic rarity, ontogenetic variability, and morphological diversity of this muscle calls for targeted investigations integrating anatomical, developmental, and functional methodologies.

### 9.1 Fetal and neonatal imaging studies

Future research should prioritize large-scale imaging analyses of fetal and neonatal lower limbs to verify and refine the proposed classification systems for FTM origin and insertion. The application of high-resolution MRI, micro-CT, and three-dimensional ultrasonography in fetuses and neonates may reveal subtle morphogenetic trajectories previously unrecognized in dissection-based studies. Longitudinal data correlating prenatal FTM presence with postnatal anatomy and function would substantially advance developmental understanding and may have implications for congenital musculoskeletal diagnostics.

### 9.2 Comparative electromyography and kinematics

Electromyographic (EMG) analyses of FTM activity during gait, eversion, and directional shifts remain scarce. Comparative studies between humans with and without FTM, as well as among non-human primates and quadrupedal mammals, could elucidate the functional emergence of this muscle in response to bipedal locomotor demands. Combined kinematic and kinetic data would help quantify the contribution of each FTT insertional variant to ankle mechanics, proprioception, and lateral stabilization. This could also inform rehabilitation strategies tailored to individual tendon morphology.

### 9.3 Biomechanical modeling and functional simulation

The use of finite element analysis (FEA) and musculoskeletal simulation platforms (e.g., OpenSim, AnyBody) may allow for prediction of strain distribution and torque transmission across the lateral foot column depending on FTM morphology. Such modeling would provide valuable insight into how different insertion patterns especially fan-shaped or bifurcated types influence eversion strength, energy expenditure, and susceptibility to injury. These tools could eventually support personalized surgical planning, particularly in athletes or patients with structural foot abnormalities.

### 9.4 Integration into educational and surgical frameworks

A key translational goal should be the integration of FTT classifications into anatomical atlases, surgical textbooks, and clinical imaging guidelines. Educational resources must reflect the spectrum of FTT variants and their implications in tendon harvesting, lateral ankle stabilization, and nerve preservation. Additionally, standardized imaging protocolsshould be developed to improve the detection and interpretation of FTT in musculoskeletal radiology, particularly in preoperative planning.

## 10 Limitations

Several limitations should be acknowledged when interpreting the current synthesis of FTM anatomy, development, and clinical relevance.

### 10.1 Population and sample size constraints

Most anatomical studies addressing FTM morphology are limited by small sample sizes, often derived from regional or ethnically homogenous populations. Consequently, the true global prevalence and variation spectrum of the FTM, especially in underrepresented groups, may remain underestimated. Similarly, comparative analyses in non-human mammals are often based on isolated taxonomic examples, limiting the phylogenetic depth and cross-species generalizability.

### 10.2 Incomplete ontogenetic and longitudinal data

While the classification systems proposed for fetal and adult FTM attachment sites offer a structured framework, there is no current longitudinal data tracking the developmental transition of FTM morphology from fetal life to adulthood. This hampers precise understanding of ontogenetic pathways, especially in relation to mechanical demands, postural maturation, or neuromuscular development.

### 10.3 Imaging and functional correlation gaps

Despite growing interest in imaging the FT, no standardized MRI or ultrasonographic protocol exists for routine identification of this muscle. Inter-observer variability in recognizing FTM especially in fusion variants or absent cases poses an obstacle for reliable data collection and clinical interpretation. Furthermore, functional electromyographic studies and kinetic analyses remain limited, reducing the evidence base for assessing the biomechanical impact of FTM variants.

### 10.4 Lack of prospective clinical studies

Current understanding of FTM’s role in lateral ankle stability, Jones fractures, and nerve assessment is primarily derived from case reports, anatomical correlations, or expert opinion. Prospective, large-scale clinical studies evaluating functional outcomes in patients with differing FTM morphologies are lacking. This restricts the translational applicability of existing anatomical knowledge to evidence-based clinical protocols.

## 11 Conclusion

The FTM is not a vestigial remnant but a dynamic, evolutionarily novel muscle, variably expressed in the human lower limb and largely absent in non-human mammals. Its presence is strongly associated with functional demands of bipedal locomotion, particularly in lateral foot stabilization and swing-phase trajectory control.

The current review demonstrates that the FT exhibits considerable anatomical diversity, both in terms of proximal origin and distal insertion, with meaningful implications for biomechanics, surgical planning, and diagnostic imaging. The proposed classification systems applicable to both fetal and adult populations offer a unified morphological framework that supports consistent anatomical education, comparative analyses, and operative documentation.

Given the functional consequences of agenesis, morphological variants, and fusion patterns, it is imperative that the FTM receives greater clinical attention. This includes its routine evaluation in radiologic protocols, incorporation into preoperative imaging interpretation, and awareness during lateral ankle or tendon harvesting procedures.

Ultimately, this synthesis highlights the FTM as a clinically and evolutionarily significant structure. Bridging insights from developmental biology, comparative anatomy, and orthopaedic relevance offers a foundation for more personalized and anatomically informed approaches in lower limb diagnostics and therapy.
